# Metabolic Heterogeneity of Cerebral Cortical and Cerebellar Astrocytes

**DOI:** 10.3390/life13010184

**Published:** 2023-01-08

**Authors:** Yuanhong Sun, Ali Winters, Linshu Wang, Kiran Chaudhari, Raymond Berry, Christina Tang, Ran Liu, Shaohua Yang

**Affiliations:** Department of Pharmacology and Neuroscience, University of North Texas Health Science Center, Fort Worth, TX 76106, USA

**Keywords:** cortex, cerebellum, astrocytes, metabolism, mitochondrial respiration, glycolysis rate, heterogeneity

## Abstract

Astrocytes play critical roles in regulating neuronal synaptogenesis, maintaining blood–brain barrier integrity, and recycling neurotransmitters. Increasing numbers of studies have suggested astrocyte heterogeneity in morphology, gene profile, and function. However, metabolic phenotype of astrocytes in different brain regions have not been explored. In this paper, we investigated the metabolic signature of cortical and cerebellar astrocytes using primary astrocyte cultures. We observed that cortical astrocytes were larger than cerebellar astrocytes, whereas cerebellar astrocytes had more and longer processes than cortical astrocytes. Using a Seahorse extracellular flux analyzer, we demonstrated that cortical astrocytes had higher mitochondrial respiration and glycolysis than cerebellar astrocytes. Cerebellar astrocytes have lower spare capacity of mitochondrial respiration and glycolysis as compared with cortical astrocytes. Consistently, cortical astrocytes have higher mitochondrial oxidation and glycolysis-derived ATP content than cerebellar astrocytes. In addition, cerebellar astrocytes have a fuel preference for glutamine and fatty acid, whereas cortical astrocytes were more dependent on glucose to meet energy demands. Our study indicated that cortical and cerebellar astrocytes display distinct metabolic phenotypes. Future studies on astrocyte metabolic heterogeneity and brain function in aging and neurodegeneration may lead to better understanding of the role of astrocyte in brain aging and neurodegenerative disorders.

## 1. Introduction

Astrocytes are the largest group of glial cells in the central nervous system (CNS), playing vital roles in brain functions, including blood–brain barrier maintenance, neuronal synaptogenesis, and neurotransmitter recycling [1,2,3]. In addition, astrocytes are active players in brain energy production, utilization, delivery, and storage [4]. Astrocytes display extraordinary morphological and functional complexity and are traditionally classified into protoplasmic and fibrous astrocytes based on their morphology and location [5]. On the one hand, protoplasmic astrocytes are relatively uniformly distributed within cortical grey matter, exhibit a spongiform profile, and are closely connected with neurons and blood vessels [1,6]. On the other hand, fibrous astrocytes are organized along white matter tracts and display elongated processes and tight interaction with oligodendrocytes and myelinated axon tracts [5].

There is increasing in vitro and in vivo evidence suggesting specialized astrocyte subtypes [7,8]. Primary astrocyte culture derived from different brain regions showed different synaptogenic potentials [9]. Neural-circuit-specialized astrocytes had been found in the hippocampus and striatum [10]. Studies of regional astrocytes RNA profiling have demonstrated that astrocytes have morphological, molecular, and physiological diversities across different brain regions, such as the cortex and hippocampus [7,11,12,13]. Astrocyte heterogeneity may not only exist inter-regionally but also intra-regionally. Astrocyte subpopulations with distinct electrophysiological and unique molecular properties have been found in the adult mouse cerebral cortex [14]. A recent single-cell in situ transcriptomic study indicates that astrocytes show both laminar and area heterogeneity across the cerebral cortex [13]. This newly discovered molecular and transcriptional diversity of astrocytes indicates that astrocytes may display metabolic heterogeneity in different states and different brain regions. Indeed, reactive astrocytes undergo morphological, molecular, functional as well as metabolic remodeling in response to CNS injury [15]. However, the potential heterogeneity of astrocyte metabolism in different brain regions has not been explored. 

The brain is one of the most energy-consuming organs with glucose as the essential fuel, consuming 25% of glucose with only 2% of the body’s weight [16]. The brain is highly complex with diverse structural characteristics in accordance with specific functions. Accordingly, differences in regional function, cellular compositions, and active metabolic pathways may link to differences in glucose metabolism in different brain regions. Positron emission tomography studies have demonstrated that the cerebral cortex has higher glycolysis than the cerebellum [17,18]. Similarly, our recent study using acute brain biopsy metabolic analysis indicates lower oxidative phosphorylation and glycolysis in the cerebellum than the other brain regions, including the cerebral cortex, hippocampus, and basal ganglia [19]. Brain function depends upon complex metabolic interactions between different cell types in which astrocytes play a central role in neuro-metabolic coupling. We expect that astrocytes may diverge between different brain regions with different metabolic phenotypes. In the current study, we developed primary astrocyte cultures from mouse cerebral cortex and cerebellum for a comprehensive analysis of their metabolic phenotype. 

## 2. Materials and Methods

### 2.1. Animals

All animal procedures were approved by the Institutional Animal Care and Use Committee of the University of North Texas Health Science Center (UNTHSC). C57BL/6J mice were purchased from the Jackson Laboratory (Bar Harbor, ME, USA) and kept in ventilating cages with access to chow and water ad libitum under a fixed 12:12 light–dark cycle. 

### 2.2. Primary Astrocyte Culture

Primary astrocytes were collected according to the published methods with minor modifications [20,21]. The primary astrocytes culture medium was composed by glucose Dulbecco’s Modified Eagle’s Medium (DMEM with 1000 mg/L glucose, 4 mM L-glutamine, 110 mg/L pyruvate, Hyclone, Smithfield, Australia) containing streptomycin (10,000 μg/mL)-Penicillin (10,000 units/mL) and 10% of heat-inactivated fetal bovine serum (FBS, Atlanta Biologicals, Flowery Branch, GA, USA). Post-natal day three to five pups (both sexes) were anesthetized by hypothermia. The cerebral cortex and cerebellum were separated and harvested in an aseptic environment. The cerebral cortex and cerebellum were transferred into a trypsin EDTA solution (1% trypsin, 0.02% EDTA) for 15-min digesting at 37 °C in a water bath. Cell suspension was strained through 40 µM strainers and centrifuged at 1200 rpm for 5 min. The cell pellets were re-suspended in the culture medium, stained by trypan blue, and the cell numbers were counted with a hemocytometer. The separated cells were seeded in poly-L-lysine precoated 100 mm culture dishes at a cell density of 3 × 10^5^/mL, and the medium was replaced 30 min later to remove the unattached cells. One half of the culture medium was replaced every three days. When cells became 90% confluent, the plates were shaken at 100 rpm for 18 h at 37 °C to eliminate microglia. All the experiments were conducted in passage 2 cortical and cerebellar astrocytes. The purity of cultured astrocytes was confirmed by immunocytochemistry and Western blot of astrocyte, neuron, microglia, and oligodendrocyte markers. 

### 2.3. ATP Assay

Cortical and cerebellar astrocytes were seeded in the 24-well plate at a density 1 × 10^5^ cells/well in 10% heat-inactivated FBS glucose DMEM medium. When the cells grew to 90% confluence, they were treated with 10 µg/mL oligomycin for 2 h. Cortical and cerebellar astrocytes were washed with PBS twice. Lysis buffer (70 μL, 500 mM Tricine buffer, pH 7.8, 2 mM EDTA, 100 mM MgSO_4_ and 2 mM sodium azide, 1% Triton X-100) were added to each well. Cell scraper was used to remove the cells, and cell lysate was collected into microcentrifuge tubes on ice. ATP reaction buffer (100 μL, 30 μg/mL D-luciferin, 25 µg/mL luciferase, and 20 µM DTT) and cell lysate (10 µL) were added to 96-well plates together with ATP standard. Luminescence was measured using a Tecan Infinite F200 plate reader. The protein content of each sample was determined simultaneously using the Pierce 660 nm protein assay (660 nm absorbance).

### 2.4. Glucose Uptake Assay

Glucose uptake was determined by glucose analog 2-NBDG as in a previous publication with minor modification [22,23]. Primary cortical and cerebellar astrocytes were seeded in 12-well plates at a density of 150,000 cells/well. After the cells reached 90% confluence, the cells were washed with glucose-free Krebs Ringer HEPES (KRH) buffer (129 mM NaCl, 5 mM NaHCO_3_, 4.8 mM KCl, 1.2 mM KH_2_PO_4_, 1 mM CaCl_2_, 1.2 mM MgCl_2_, 10 mM HEPES; pH 7.4) twice and incubated in glucose-free KRH buffer for 30 min. After 30-min starvation, the same volume of glucose-free KRH buffer containing 100 μM 2-NBDG was added to each well. After 2 min incubation, cells were washed with KRH buffer twice. Each well was filled with 400 µL RIPA buffer (50 mM of Tris. HCl, pH 7.4, 150 mM NaCl, 1 mM EDTA, 1% Triton X 100). Cell scraper was used to remove the cells, and cell lysate was collected into the microcentrifuge tubes. The cell lysate was centrifuged at 12,000 rpm for 5 min at 4 °C. The supernatant was collected. 100 μL supernatant of each sample was added into a black 96-well plate. Fluorescent was determined by a Tecan Infinite F200 plate reader with excitation and emission wavelengths at 465 nm and 540 nm, respectively. The protein concentration of each sample was determined using the Pierce 660 nm protein assay simultaneously (660 nm absorbance). The concentration of intracellular 2-NBDG was normalized to the protein concentration.

### 2.5. Extracellular Glutamate Clearance

Primary astrocytes were seeded in 24-well plates at 5 × 10^4^ cells/well in the culture medium. When the cells reached 80% confluence, the culture media were replaced with glutamate elimination medium (DMEM with 5.5 mM glucose and 2% FBS). After 12-h incubation, the medium was removed, and plates were washed twice with glutamate uptake buffer (1.3 mM CaCl_2_, 1.2 mM KH_2_PO_4_, 10 mM D-glucose, 122 mM NaCl, 0.4 mM MgSO_4_, 2.2 mM KCl, 25 mM HEPES, pH 7.4). Subsequently, 500 µL uptake buffer containing 30 µM glutamate was immediately added to each well. Glutamate uptake buffer was collected at 30, 60, 90, and 120 min after glutamate addition. RIPA lysis buffer (70 µL) was added simultaneously to each well at different time points after glutamate uptake buffer collection, and cell lysate was collected in the microcentrifuge tubes. Extracellular glutamate concentrations were determined and analyzed according to the manufacturer’s protocol (Amplex Red Glutamic acid/Glutamate Oxidase assay kit, Life Technologies, Carlsbad, CA, USA). The protein content of each sample was determined using the Pierce 660 nM protein assay (660 nM absorbance). Data are presented as the extracellular glutamate concentration.

### 2.6. Growth Curve

Cortical and cerebellar astrocytes were seeded in 12-well plates precoated with poly-L-lysine at a density of 5 × 10^4^ cells/well in a culture medium. The day for seeding the cells was set as day 0, and cortical and cerebellar astrocytes were harvested at day 3 to day 6 using trypsin-EDTA and were counted with a hemocytometer. Cell counting was performed by a different researcher who was blinded to the groups using a Zeiss Invertoskop microscope. 

### 2.7. Immunocytochemistry

Cortical and cerebellar astrocytes were seeded on poly-L-lysine pre-coated coverslips in 24-well plates at a density of 3 × 10^4^ cells/well in the culture medium. When they reached 70% confluence, the cells were fixed with 10% neutral buffered formalin for 15 min at room temperature. Cortical and cerebellar astrocytes were permeabilized with PBST (containing 0.2% Trion X, pH 7.4). Cells were blocked with 3% donkey serum in superblock buffer for 1 h at room temperature and incubated with the following primary antibodies overnight at 4 °C (anti-GFAP, 1:200, Cell Signaling Technology, Cat #3670S; anti-Iba1, 1:100, Cell Signaling Technology, Cat #17198; anti-ALDH1L1, 1:100, Abcam, Cat #87117). The cells were then incubated with secondary antibodies (Alex-Fluor^TM^ 488 donkey anti-mouse IgG, 1: 500, Cat A21202; Alex-Fluor^TM^ 568 donkey anti-rabbit IgG, 1: 500, Cat: # A10042) for 60 min at room temperature and mounted with ProLong^TM^ Gold antifade reagent with DAPI (Lot #1899752, Invitrogen, Waltham, MA, USA). Fluorescence and differential interference contrast (DIC) images were taken with a Zeiss Axio Observer Z1 microscope (Carl Zeiss, Dublin, CA, USA). The morphologies of cortical and cerebellar astrocytes were analyzed using FIJI-ImageJ (RRID:SCR_002285, NIH, Bethesda, MD, USA), and a Simple Neurite Tracer (SNT) plugin [24].

### 2.8. Western Blot

Cortical and cerebellar astrocytes were seeded at a density of 3 × 10^5^ cells/well in 6-well plates in the culture medium. When primary astrocytes reached 90% confluence, RIPA lysis buffer (50 mM of Tris-HCl, pH7.4, 1 mM EDTA, 150 mM NaCl, 1% Triton X 100 with phosphatase and protease inhibitors) was used to lyse the cells. The protein concentrations in the supernatants were determined using the Pierce 660 nm protein assay (Thermo Fisher Scientific, Waltham, MA, USA). The protein samples were resolved on SDS-PAGE (sodium dodecyl sulfate-polyacrylamide gel electrophoresis) and transferred to a PVDF membrane. The membranes were blocked with 5% non-fat milk for 1 h at room temperature. The membranes were probed with the following primary antibodies at 4 °C overnight (anti-vimentin, 1:1000, Cell Signaling Technology, Cat #5741S; anti-ALDH1L1, 1:1000, Abcam, Cat #87117; anti-GFAP, 1:1000, Cell Signaling Technology, Cat #12389; anti-MAP2, 1:1000, Cell Signaling Technology, Cat #8707S; anti-MBP, 1:5000, Novus Biologicals, Cat #50035; anti-GAPDH, 1:5000, Santa Cruz Technology, Cat #sc-3223; anti-β-actin, 1:5000 dilutions, Santa Cruz Technology, Cat #47778). The membranes were incubated with appropriate secondary antibodies labeled with horseradish peroxidase (Jackson immunoResearch Laboratories, West Grove, PA, USA). A chemiluminescence signal was detected with a Biospectrum 500 UVP imaging system. FIJI-ImageJ (NIH, Bethesda, MD, USA) was used to quantify the target protein bands’ density and normalized to β-actin or GAPDH.

### 2.9. Seahorse Analysis

For metabolic analysis, cortical and cerebellar astrocytes were seeded into a Seahorse XFe 96 culture microplate at a density of 20,000 cells/well. One day prior to the experiment, the sensor cartridge was hydrated in deionized H_2_O in a non-CO_2_ incubator at 37 °C overnight. On the day of the experiment, deionized H_2_O was replaced with Seahorse calibrant in the sensor cartridge and placed in a non-CO_2_ incubator at 37 °C for 1 h. The culture media were replaced with Seahorse XF base medium containing 1 mM pyruvate, 2 mM glutamine, and 10 mM glucose in the XFe96 culture microplate and incubated in a non-CO_2_ incubator at 37 °C for 1 h. Real-time metabolic analysis was conducted using a Cell Energy Phenotype Test kit, Mito Stress Test kit, Glycolytic Rate Assay kit, and Mitochondrial Fuel Flex test kit (Agilent Technologies, Santa Clara, CA, USA). All measurements were normalized to the cell number by using Hoechst staining.

Mito Stress and Cell Energy Phenotype assays: oligomycin, carbonyl cyanide-4-(trifluoromethoxy) phenylhydrazone (FCCP), and rotenone/antimycin A (Rot/AA) were diluted in a Seahorse XF base medium and loaded into the cartridges to acquire the final concentration of 1.5, 2.0, and 0.5 µM, respectively. Oligomycin, FCCP, and Rot/AA were injected into the well sequentially. Mito Stress Test parameters were monitored with each cycle setting as the mix for 150 s, delay for 30 s, and then measured for 60 s, with four cycles.

Glycolysis Rate assay: Rot/AA and 2-deoxyglucose (2-DG) were diluted in the XF base medium and loaded into the cartridges to obtain the final concentrations of 0.5 µM and 50 mM. Rot/AA and 2-DG were injected into the well sequentially. Glycolytic rate assay parameters were monitored with each cycle setting as the mix for 150 s, delayed for 30 s, and measured for 60 s, with three–five cycles. 

Mito Fuel Flex test: Three different inhibitors with different combinations were used to measure astrocytes’ dependency, capacity, and flexibility of glucose (GLC), glutamine (GLN), and fatty acids (FAs). The three inhibitors used were: UK5099 (an inhibitor of mitochondrial pyruvate carrier protein, MPC) was used to inhibit glucose/pyruvate metabolism through mitochondrial respiration. BPTES (an allosteric inhibitor of glutaminase, GLS1) was used to inhibit the glutamine/glutamate oxidation pathway. Etomoxir was used to inhibit the carnitine palmitoyl-transferase 1A and block the translocation of long-chain fatty acids from the cytosol into the mitochondria for β-oxidation. By sequentially adding different inhibitors of each fuel and measuring the variant rate of oxidation respiration, each substrate’s dependency, capacity, and flexibility could be calculated. UK5099, BPTES, and etomoxir, R/A were diluted in the XF base medium and loaded into the cartridges in order to obtain the final concentration of 2 µM, 3 µM, and 4 µM, 0.5 µM. Different combinations of UK5099, BPTES and etomoxir were injected into the well sequentially, with the last injection of R/A. Mito Fuel Flex test parameters were monitored with each cycle setting as the mix for 150 s, delayed for 30 s and then measured for 60 s, with three–six cycles. 

### 2.10. Statistical Analysis

Statistical analysis was processed with GraphPad Prism software. All the results were presented as mean ± standard deviation (SD). To determine the cortical and cerebellar astrocytes’ morphology, 12 to 20 astrocytes were used to compare the cellular area, number of processes, and length of process. An unpaired *t*-test was used to identify any significant difference. The Mito Stress Test, Glycolytic Rate Assay, and Mito Fuel Test were performed by the Seahorse XFe96 analyzer and data were compared using an unpaired *t*-test. A minimum of seven individual wells were involved in each experimental group and each experiment was repeated in three independent cultures. Representative results were shown in the results. A two-way ANOVA with Tukey’s multiple comparisons was used to compare the data sets in the ATP assay (the first factor is regional astrocytes, which have two levels of cortical astrocytes and cerebellar astrocytes; the second factor is the different treatment which contains two levels of oligomycin treatment and no treatment) and a two-way ANOVA with Sidak’s multiple comparisons test was used to compare data sets in the glutamate clearance (the first factor is regional astrocytes, which have two levels of cortical astrocytes and cerebellar astrocytes; the second factor is the different time point which contains three levels of 30 min treatment, 60 min treatment, 120 min treatment) and cell proliferation (the first factor is regional astrocytes which have two levels of cortical astrocytes and cerebellar astrocytes; the second factor is the different time point, which contains four levels of three days, four days, five days, and six days). Effect size (Cohen’s d and Cohen’s f) was performed. A *p*-value < 0.05 was considered statistically significant.

## 3. Results

**Figure 1:**
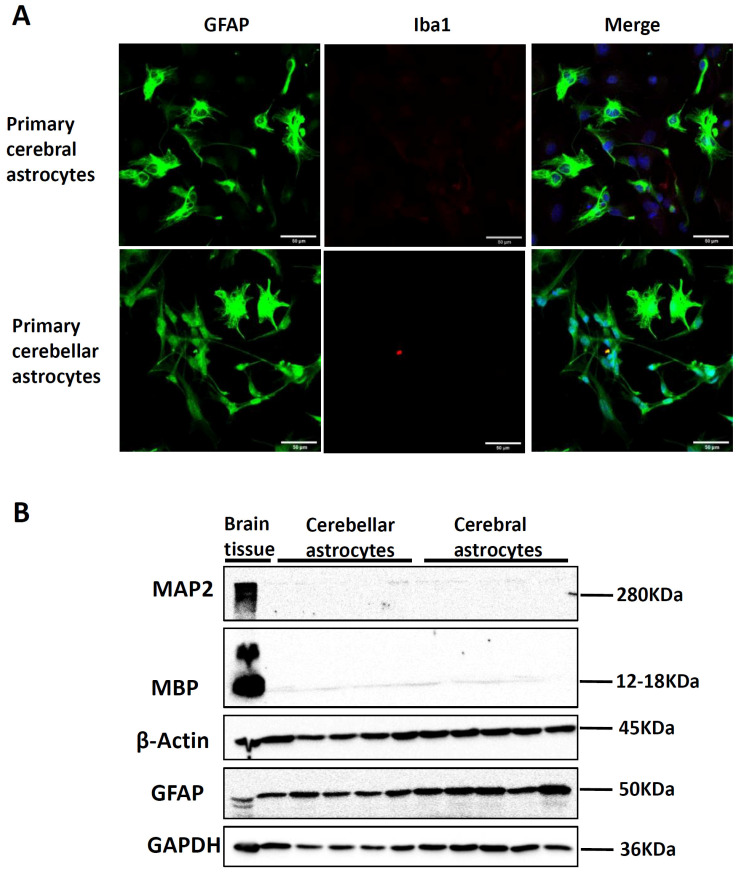
Investigation of primary cortical astrocytes (CX) and cerebellar astrocytes (CE) purification. (**A**) Representative image of GFAP and Iba1 immunostaining of cortical astrocytes and cerebellar astrocytes, scale bar = 20 μm. (**B**) Western blots of MAP2, MBP, β-actin, GAPDH of cortical astrocytes and cerebellar astrocytes.

The purity of primary astrocyte culture was assessed by immunocytochemical staining and Western blot (Figure 1). Immunocytochemistry staining results showed that most cells were stained positive for the astrocyte marker GFAP, and very few cells were stained positive for the microglia cell marker Iba1 (Figure 1A). Western blot results further demonstrated that cortical and cerebellar astrocyte cultures were enriched with astrocytes without neuron or oligodendrocyte contamination (Figure 1B).

### 3.1. The Cerebral Cortical and Cerebellar Astrocytes Display Distinct Morphologies

**Figure 2:**
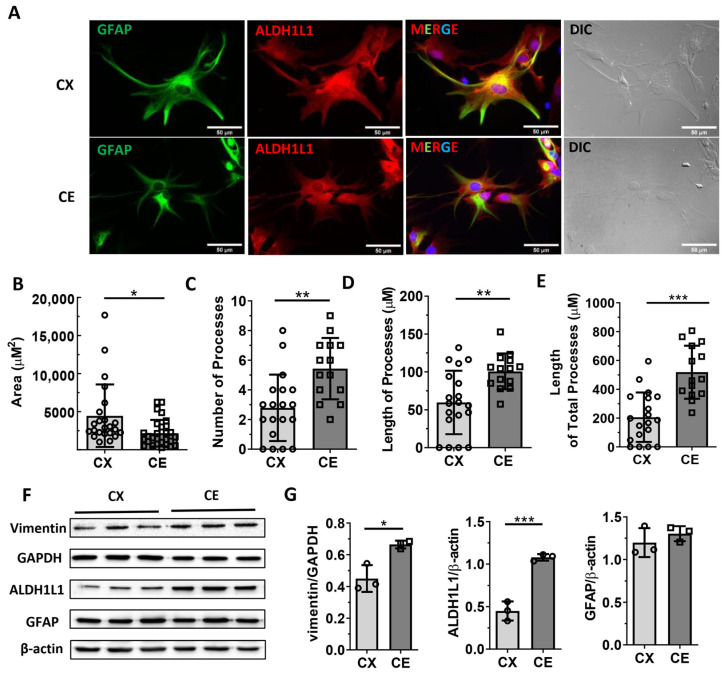
The morphology and astrocytic markers expression of cortical astrocytes and cerebellar astrocytes. (**A**) Representative immunocytochemistry images of GFAP and ALDH1L1 in primary cortical astrocytes and cerebellar astrocytes, scale bar = 50 µm. (**B**–**E**) Quantitative analysis of cell area, number of processes, and length of processes in cortical and cerebellar astrocytes (*n* = 12–20). (**F**) Western blot results of vimentin, AlDH1L1, and GFAP expression in primary cortical astrocytes and cerebellar astrocytes. The uncropped blots and molecular weight markers are shown in Supplementary Materials. (**G**) Quantitative analysis of vimentin, ALDH1L1, and GFAP expression level in primary cortical astrocytes and cerebellar astrocytes (n = 3). Circle and square represent values of individual data point. Unpaired *t-*test, * *p* < 0.05, ** *p* < 0.01, *** *p* < 0.0001, Data are expressed as mean ± SD.

The immunocytochemistry demonstrated that both cortical and cerebellar astrocytes were positively stained with GFAP and ALDH1L1 with a different pattern. ALDH1L1 labeled both the cell bodies and processes of primary astrocytes, while GFAP only labeled the extensive processes and a subset cell body of astrocytes (Figure 2A). Therefore, we chose ALDH1L1 and GFAP staining for cell area and cell processes quantification, respectively (Figure 2B*–*E). Compared with cortical astrocytes, cerebellar astrocytes have smaller cell areas and more processes (Figure 2B,C; t_49_ = 2.6680, Cohen’s d = 0.7373, *p* = 0.0103; t_31_ = 3.4460, Cohen’s d = 1.2687, *p* = 0. 0017). In addition, the total and average length of processes was longer in cerebellar astrocytes as compared with cortical astrocytes (Figure 2D,E; t_31_ = 3.2750, Cohen’s d = 1.2006, *p* = 0.0026; t_31_ = 5.019, Cohen’s d = 1.7592, *p* < 0.0001). Western blot results showed higher expression levels of vimentin and ALDH1L1 in cerebellar astrocytes as compared with cortical astrocytes (Figure 2F,G; t_4_ = 4.2830, Cohen’s d = 3.4967, *p* = 0.0128; t_4_ = 9.1290, Cohen’s d = 7.4597, *p* = 0.0008). The cortical and cerebellar astrocytes expressed a similar level of GFAP (Figure 2G). 

### 3.2. Mitochondrial Respiration Function and Bioenergetic Phenotype of Cortical and Cerebellar Astrocytes

**Figure 3:**
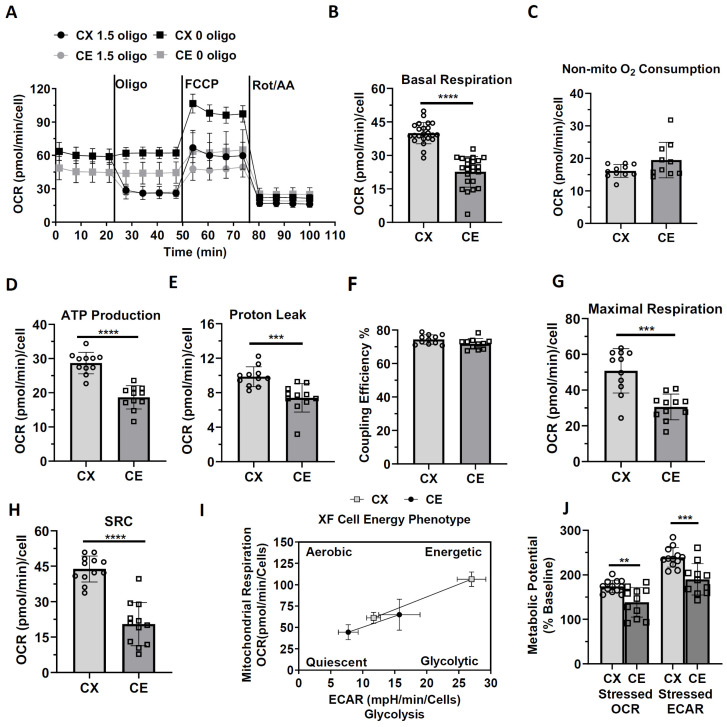
Mitochondrial respiration and cell energy phenotype of primary cortical astrocytes and cerebellar astrocytes. Experiments were performed by using a Seahorse Mito-Stress kit in XFe96 extracellular flux analyzer as detailed in the materials and methods. (**A**) The traces of oxygen consumption rates before and after adding the following compounds to the assay micro-chambers: oligomycin (oligo.), carbonylcyanide-4-(trifluoromethoxy) phenylhydrazone (FCCP), rotenone plus antimycin A (Rot/AA). n = 11–23. (**B**) Scatter plot and bar graph of basal respiration. n = 23. (**C**) Scatter plot and bar graph of non-mitochondrial O_2_ consumption. n = 10–11. (**D**) Scatter plot and bar graph of ATP production. n = 11. (**E**) Scatter plot and bar graph of proton leak-associated respiration. n = 11. (**F**) Scatter plot and bar graph of coupling efficiency between primary cortical astrocytes and cerebellar astrocytes. n = 11. (**G**) Scatter plot and bar graph of maximal respiration. n = 11. (**H**) Scatter plot and bar graph of spare respiration capacity (SRC). n = 12. (**I**) Energy phenotype of cortical astrocytes and cerebellar astrocytes with or without stress stimulation. n = 12–46. (**J**) Quantitative analysis of the metabolic potential of cortical astrocytes and cerebellar astrocytes. n = 11–12. Circle and square represent values of individual data point. Unpaired *t*-test, ** *p* < 0.01, *** *p* < 0.001, and **** *p* < 0.0001, Data are expressed as mean ± SD.

We determined the metabolic phenotype of cortical and cerebellar astrocytes using a Seahorse XFe 96 Mito Stress Test. Mitochondrial complex V inhibitor oligomycin had been reported to suppress the OCR response after FCCP treatment; hence, it led to underestimating the maximal electron-transported system capacity [25]. Therefore, oligomycin omitted groups were included to acquire the maximal respiration after uncoupler FCCP treatment (Figure 3A). Compared with cortical astrocytes, cerebellar astrocytes showed a significantly lower basal OCR (Figure 3B, t_44_ = 9.9620, Cohen’s d = 2.9366, *p* < 0.0001). No significant difference in non-mitochondrial associated OCR was observed between cortical and cerebellar astrocytes (Figure 3C). Cortical astrocytes showed significantly higher ATP production and proton leak-linked respiration than cerebellar astrocytes (Figure 3D,E; t_20_ = 7.1990, Cohen’s d = 3.0690, *p* < 0.0001; t_20_ = 4.0400, Cohen’s d = 1.7192, *p* = 0.0007). Similar coupling efficiency was observed between cortical astrocytes and cerebellar astrocytes (Figure 3F). Significantly lower maximal respiration and spare capacity were indicated in cerebellar astrocytes than in cortical astrocytes (Figure 3G,H; t_20_ = 4.6803, Cohen’s d = 1.9952, *p* = 0.0001; t_22_ = 7.5803, Cohen’s d = 3.0943, *p* < 0.0001). Assessment of metabolic phenotypes suggested that both cortical astrocytes and cerebellar astrocytes showed more energetic phenotypes in the stressed situations (Figure 3I). Compared with cortical astrocytes, cerebellar astrocytes presented a more quiescent phenotype due to the lower aerobic and glycolytic-related metabolic potential in response to stressed conditions (Figure 3J, t_21_ = 3.3853, Cohen’s d = 1.3894, *p* = 0.0028; t_21_ = 4.0783, Cohen’s d = 1.6867, *p* = 0.0005). Cortical astrocytes showed a higher glycolytic metabolic potential compared to cerebellar astrocytes (Figure 3J).

### 3.3. Glycolytic Function of Cortical and Cerebellar Astrocytes

**Figure 4:**
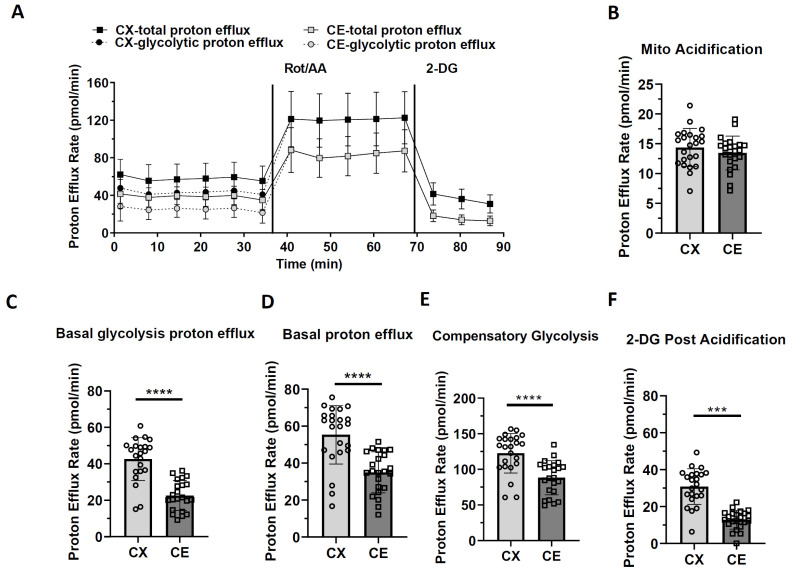
Glycolytic function analysis of primary cortical astrocytes and cerebellar astrocytes. Experiments were performed by using Seahorse Glycolytic Rate kit in XFe96 extracellular flux analyzer as detailed in the materials and methods. (**A**) The traces of proton efflux rates before and after adding Rot/AA and 2-deoxy glucose (2-DG) to the assay micro-chambers. (**B**) Scatter plot and bar graph of Mitochondrial acidification (mito acidification). n = 23. (**C**) Scatter plot and bar graph of basal glycolysis proton efflux rate. n = 22. (**D**) Scatter plot and bar graph of basal proton efflux. rate. n = 23. (**E**) Scatter plot and bar graph of compensatory glycolysis proton efflux rate. n = 23. (**F**) Bar graph of 2-DG post acidification. n = 23. Results of proton efflux rate curve and bar graph are shown as mean ± SD. Unpaired *t*-test, *** *p* < 0.001, **** *p* < 0.0001.

We further investigated the glycolytic function of cortical and cerebellar astrocytes. The proton efflux rate was calculated according to OCR and ECAR values. After eliminating the mitochondrial respiration produced CO_2_-contributed proton efflux rate, the glycolytic-associated proton efflux rate could be more accurately calculated and utilized to reflect the glycolytic function of cortical and cerebellar astrocytes (Figure 4A). The cerebellar astrocytes showed a similar mitochondrial respiration-associated proton efflux rate, but a lower basal glycolysis-associated proton efflux rate, as compared with cortical astrocytes (Figure 4B,C; t_42_ = 6.5470, Cohen’s d = 1.9805, *p* < 0.0001). Overall, cortical astrocytes had a significantly higher basal proton efflux rate than cerebellar astrocytes (Figure D; t_44_ = 5.0520, Cohen’s d = 1.4896, *p* < 0.0001). A higher compensatory glycolysis proton efflux rate was found in cortical astrocytes than in cerebellar astrocytes, implying that cortical astrocytes had a higher glycolytic function to meet the energy demands during stress conditions (Figure 4E; t_44_ = 4.4870, Cohen’s d = 1.3230, *p* < 0.0001). After we added 2-DG, a higher post-2-DG associated proton efflux rate was also observed in cortical astrocytes than in cerebellar astrocytes, suggesting that cortical astrocytes could utilize the other fuel for glycolysis beside glucose (Figure 4F; t_44_ = 7.9010, Cohen’s d = 2.3295, *p* < 0.0001).

### 3.4. Metabolic Dependency and Flexibility of Cortical and Cerebellar Astrocytes

**Figure 5:**
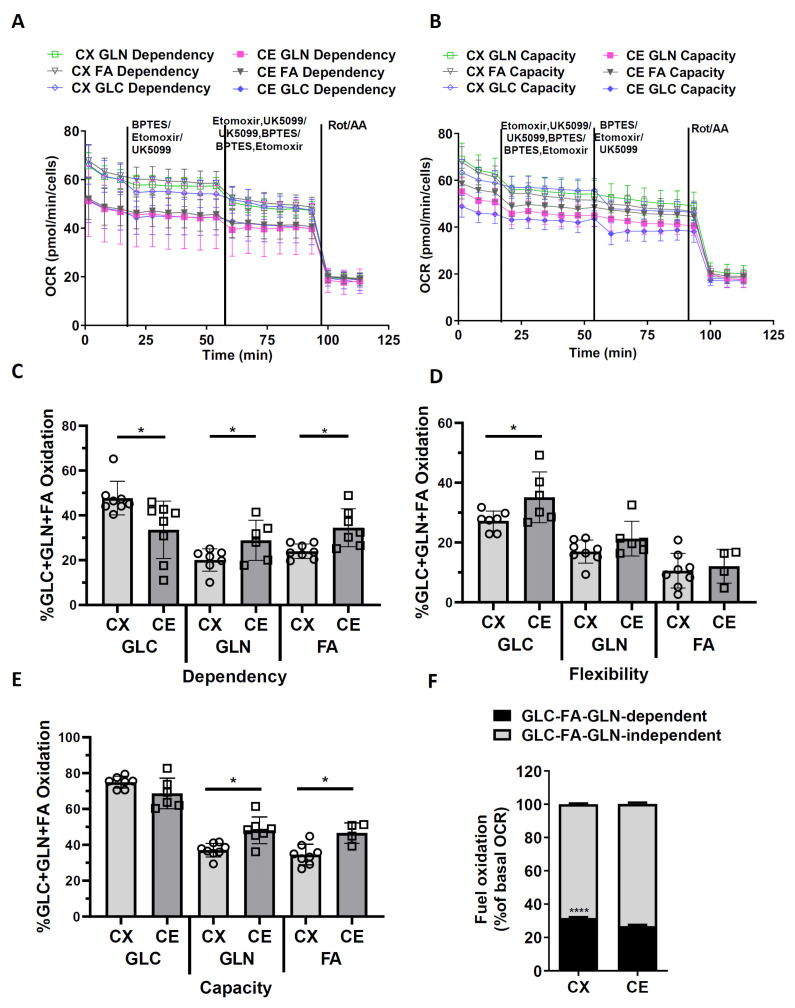
Mitofuel assay of primary cortical astrocytes and cerebellar astrocytes. Experiments were performed by using a Seahorse Mito-Fuel kit in XFe96 extracellular flux analyzer as detailed in the materials and methods. (**A**,**B**) are the traces of OCR of cortical astrocytes and cerebellar astrocytes in the absence of and in the presence of a cocktail of following inhibitors, where indicated the glutamine (GLN), fatty acids (FA), glucose (GLC) dependency and capacity respectively. UK5099: inhibitor of mitochondrial pyruvate carrier; etomoxir: inhibitor of glutamine; BPTES: inhibitor of carnitine palmitoyl transferase 1. Results of OCR curves are shown as mean ± SD. (**C**) Scatter plot and bar graph of GLC dependency, GLN dependency, and FA dependency. n = 6–8. (**D**) Scatter plot and bar graph of GLC flexibility, GLN flexibility, and FA flexibility. n = 4–8. (**E**) Scatter plot and bar graph of GLC capacity, GLN capacity, and FA capacity. n = 6–8. Results of bar graph are shown as mean ± SD. Unpaired *t-*test, * *p* < 0.05. (**F**) Stacked bar graph of the GLC/FA/GLN-dependent and GLC/FA/GLN-independent OCRs as percentage of Rot/AA sensitive OCR. Results of stacked bar graph are shown as mean ± SD. n = 45–46. **** *p* < 0.0001 in two-way ANOVA with a Sidak’s multiple comparisons test.

The mitochondrial Fuel Flex Test was performed to evaluate the dependency, capacity, and flexibility of different substrates (glucose GLC, fatty acids FAs, glutamine GLN) of cortical and cerebellar astrocytes. Compared with cerebellar astrocytes, a higher glucose dependency was observed in cortical astrocytes, whereas higher glucose flexibility was observed in cerebellar astrocytes (Figure 5A*–*D, t_14_ = 2.6990, Cohen’s d = 1.3497, *p* = 0.0173; t_11_ = 2.2760, Cohen’s = 1.2232, *p* = 0.0438). In addition, cortical and cerebellar astrocytes showed a similar glucose capacity (Figure 5E). The cerebellar astrocytes showed a higher dependency and capacity on fatty acids and glutamine than cortical astrocytes (Figure 5C,E, t_11_ = 2.2340, Cohen’s d = 1.2129, *p* = 0.0472; t_13_ = 3.2640, Cohen’s d = 1.6400, *p* = 0.0062; t_13_ = 3.683, Cohen’s d = 1.8630, *p* = 0.0028; t_10_ = 3.417, Cohen’s d = 2.1007, *p* = 0.0066). No significant difference in fatty acids and glutamine flexibility was observed between cortical and cerebellar astrocytes (Figure 5D). To evaluate the contribution of these three fuels to basal respiration, we measured the basal oxygen consumption rate by adding the Rot/AA to block the mitochondrial respiration completely. The cortical astrocytes showed a higher GLC-FAs-GLN pathway dependency during basal oxygen respiration than cerebellar astrocytes (Figure 5F, F_1,179_ = 66.0000, Cohen’s f = 0.5993, *p* < 0.0001).

### 3.5. Assessment of the ATP Content, Glucose, and Glutamate Uptake of Cortical and Cerebellar Astrocytes

**Figure 6:**
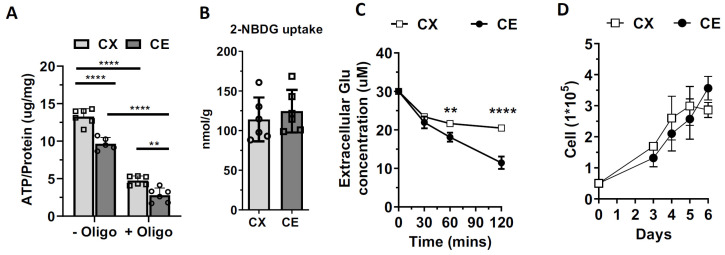
Cortical astrocytes have higher ATP content than cerebellar astrocytes. (**A**) Scatter plot and bar graph of total ATP content assay before and after adding oligomycin to the cortical astrocytes and cerebellar astrocytes. Results are shown as mean ± SD. n = 6. ** *p* < 0.01, **** *p* < 0.0001 in two-way ANOVA followed by Tukey’s multiple comparisons test. (**B**) Quantitative analysis of 2-NBDG uptake assay between cortical astrocytes and cerebellar astrocytes. Results are shown as mean ± SD. n = 6. Unpaired *t-*test. (**C**) Extracellular glutamate uptake assay. Results are shown as mean ± SD. n = 4. ** *p* < 0.01, **** *p* < 0.0001 in one-way ANOVA with a Sidak’s multiple comparisons test. (**D**) Growth curve assay of cortical astrocytes and cerebellar astrocytes. Results are shown as mean ± SD. n = 4. Two-way ANOVA with a Sidak’s multiple comparisons test.

We measured the ATP content in cortical and cerebellar astrocytes. A higher ATP level was observed with or without oligomycin treatment in cortical astrocytes than cerebellar astrocytes (Figure 6A, F_1,19_ = 5.2090, Cohen’s f = 0.4477, *p* = 0.0342). These results indicated that a higher ATP level in cortical astrocytes was attributed to both the glycolytic process and mitochondrial respiration. To investigate if higher mitochondrial respiration and glycolytic function were associated with glucose intake, we performed a 2-NBDG uptake assay; no significant difference in 2-NBDG uptake was observed between cortical and cerebellar astrocytes (Figure 6B). We further investigated glutamate uptake ability in cortical and cerebellar astrocytes. Cerebellar astrocytes showed a significantly higher glutamate clearance capacity than cortical astrocytes (Figure 6C; F_3,16_ = 244.4000, Cohen’s f = 6.0374, *p* < 0.0001). In addition, no significant difference in the proliferation rate was observed between cortical astrocytes and cerebellar astrocytes (Figure 6D).

## 4. Discussion

There are three major findings derived from the current study using primary astrocyte cultures. First, the cortical and cerebellar astrocytes display unique morphology with different cell size, number of processes, and expression level of ALDH1L1 and vimentin. Second, cerebellar astrocytes have an overall more quiescent metabolic phenotype with a lower basal and spare mitochondrial respiration and glycolysis as compared with cortical astrocytes. Third, cortical and cerebellar astrocytes have different metabolic substrate preferences. The cerebellar astrocytes have a higher glutamine and fatty acid dependency and lower glucose dependency as compared with cortical astrocytes.

Astrocytes, the most abundant cells in the CNS, play an extensive role in maintaining the brain’s physiological functions. Increasing numbers of studies have demonstrated that morphological and molecular diversity exists between and within brain regions [11,26]. Transcriptionally and morphologically distinct astrocytes in the mouse cortex and hippocampus have been demonstrated in a single RNA sequencing study. In the present study, our results showed that cortical astrocytes and cerebellar astrocytes have different morphologies. Compared with cortical astrocytes, more processes are observed in cerebellar astrocytes. This is consistent with a previous publication that showed that more processes are observed in primary cerebellar astrocytes than in hippocampus and cerebral cortical astrocytes [27]. The mammalian brain is characterized by exceptionally high metabolic activity with tight neurovascular and neuroglial coupling to ensure adequate energy supply in register with neuronal activity [4,28]. Glucose is almost completely metabolized through oxidative phosphorylation in the CNS. Nevertheless, oxidative or glycolytic glucose metabolism may take place in different cell compartments in the brain. Metabolically, neurons are predominantly oxidative whereas astrocytes derive energy from both oxidative and glycolytic pathways [2]. Astrocytes, as components of a neurovascular unit, are critical for the coupling between neuronal activity and energy metabolism [29]. Nuclear magnetic resonance (NMR) results suggest the upregulation of the lactate level in the visual cortex after stimulation [30,31]. A coupling of neuronal activation and glucose utilization in astrocytes has been demonstrated [32]. Accordingly, an astrocyte–neuron lactate shuttle (ANLS) hypothesis has been proposed according to which astrocytes support neuron activity by upregulation glycolysis and releasing lactate into extracellular space to support the energy demands of neurons [33,34]. However, astrocyte function and metabolism may not be uniform across the whole brain. Astrocytes may display metabolic heterogeneity to adapt to anatomical and functional diversity in different brain regions with different cellular components.

The mammalian brain is characterized by the evolutionally expanded cerebrum and a remarkable cognitive capacity, whereas the cerebellum is considered to be a more archaic brain structure for motor coordination [35]. The fractional distribution of neurons in the brain does not correspond to the fractional distribution of mass in the brain structure. In the human brain, the cerebral cortex comprises over 80% of the brain mass but contains less than 20% of all brain neurons. In contrast, the cerebellum represents only 10% of the total brain mass but contains 80% of the brain’s neurons [36]. Furthermore, the cerebral cortex and cerebellum have distinct neuronal components. The cortical neurons are primarily glutamatergic pyramidal neurons and GABA-containing interneurons [37], while more than 99% of cerebellar neurons are glutamatergic granule cells [38]. By using the Seahorse analyzer, a recent study indicated that mouse cortical astrocytes showed higher mitochondrial respiration than human cortical astrocytes, and different glucose utilization was observed between mouse and human astrocytes [39]. The results suggested that species-dependent metabolism existed in astrocytes [39]. In the present study, we found that cerebral cortical and cerebellar astrocytes have distinct metabolic phenotypes evidenced by the Seahorse extracellular flux analysis. For glucose metabolism, cerebral cortical astrocytes have significantly higher oxidative phosphorylation and glycolysis than cerebellar astrocytes under basal and stress conditions. In addition, cerebellar astrocytes display significantly lower spare capacity of mitochondrial respiration and glycolysis. The lower oxidative phosphorylation and glycolysis of cerebellar astrocytes is further evidenced by their lower ATP content as compared with cortical astrocytes. No difference was found in 2-NBDG uptake between cortical and cerebellar astrocytes. Thus, the different glucose metabolic phenotypes between cortical and cerebellar astrocytes is likely due to the different intracellular metabolic pathways in cortical and cerebellar astrocytes. Furthermore, our study indicates that cortical and cerebellar astrocytes may have a different preference and capability to use different substrates for mitochondrial respiration.

Besides glucose, astrocytes use different substrates, such as glutamate and fatty acid, to meet their energy requirements [40]. In the present study, we conducted a Mito fuel flex test to assess astrocytes’ dependency and the flexibility of glucose, glutamine, and fatty acids metabolism. Fuel dependency measures the cells’ reliance on a fuel pathway to maintain basal respiration. Fuel capacity evaluates the ability of cells’ to use a fuel to meet basal energy requirements when oxidation of the other two substrates was blocked. Fuel flexibility reflects the cells’ ability to switch or compensate mitochondrial respiration from one substrate to another [41]. Our results indicated that cortical astrocytes are more dependent on glucose for fuel supply than cerebellar astrocytes, while cerebellar astrocytes are more flexible on different fuel and rely more on glutamine and fatty acid for mitochondrial respiration. In addition, cerebellar astrocytes have a higher capacity for glutamine and fatty acid oxidation than cortical astrocytes.

Glutamate is the main excitatory neurotransmitter of glutamatergic neurons [42]. Astrocytes play a critical role in glutamate recycling through the glutamate–glutamine cycle [42]. Glutamate is converted to glutamine through glutaminase in astrocytes and released back into the tripartite synapse for uptake by neurons [43]. In addition, glutamate can be metabolized through the Krebs cycle and oxidative phosphorylation in astrocytes to offset the high ATP cost of glutamate uptake [44]. Glutamatergic granule cells comprise over 99% of cerebellar neurons [38,45]. The neuron/glia ratio is dramatically different between the cerebral cortex and cerebellum at ~1.5 and ~0.2, respectively [36]. Thus, cerebellar astrocytes have a much higher burden for glutamate recycling to maintain a low extracellular glutamate concentration to protect granule cells from excitotoxicity [46,47]. A previous study demonstrated that primary mouse cerebellar astrocytes had a higher glutamine uptake ability than cerebral cortical astrocytes [48]. Interestingly, the Vmax of glutamate uptake, when measured within 30 min of glutamate treatment, was lower in primary rat cerebellar astrocytes than in cortical astrocytes [49]. We determined glutamate uptake capacity in primary astrocytes at 30, 60, and 120 min after glutamate treatment. We observed no significant difference in the amount of glutamate uptake at 30 min. However, cerebellar astrocytes uptake significantly more glutamate than cortical astrocytes at 60 and 120 min. Given their high fuel dependency on glutamine, high fuel flexibility in glucose, and high glutamate uptake capacity, cerebellar astrocytes likely have high glutamate oxidation for ATP production to adapt to their microenvironment of extreme high glutamatergic neuron density.

In this study, we demonstrated that heterogeneous metabolism existed between mouse cortical astrocytes and the cerebellar. However, the limitations of this study should also be made clear. First, compared with the complexity of the brain environment, primary astrocytes lack cell–cell interactions, which may modify the astrocytes’ metabolism. In addition, we used primary cultured astrocytes collected from young pups. Therefore, adult and/or aging mouse regional astrocytes’ metabolic characteristics could not be directly established without further investigation. In the future, by taking advantage of our lab’s recently optimized brain tissue Seahorse analyzer protocol, we will study the regional astrocytes’ metabolism under different conditions on the tissue level [19].

## 5. Conclusions

Astrocyte heterogeneity has long been recognized in terms of morphology and function within the same region and across different brain regions. Our study discovered metabolic heterogeneity of astrocytes between the cerebral cortex and cerebellum. In the present study, we demonstrated that cerebral cortical and cerebellar astrocytes have distinct metabolic phenotypes. Compared to cortical astrocytes, cerebellar astrocytes had a less energetic phenotype with lower mitochondrial respiration and glycolysis under basal and stressed conditions. Cortical astrocytes display higher fuel dependency on glucose, whereas cerebellar astrocytes have higher fuel flexibility in glucose and higher fuel dependence on glutamine and fatty acid as compared with the former. In addition, cerebellar astrocytes have lower spare capacity of mitochondrial respiration and glycolysis as compared with cortical astrocytes. The identified metabolic heterogeneity of astrocytes offers novel insights into functional diversity and the cell-type-specific landscape of different brain regions under physiological and pathological conditions. Future studies on astrocyte metabolic heterogeneity and the brain function in aging and neurodegeneration may lead to better understanding of the role of astrocytes in brain aging and neurodegenerative disorders.

## Data Availability

The data that support the findings of this study are available from the corresponding author, S.Y., upon reasonable request.

## Supplementary Materials

The following supporting information can be downloaded at: https://www.mdpi.com/article/10.3390/life13010184/s1, File S1: Original western blots.

## References

[1] Khakh B.S., Deneen B. (2019). The Emerging Nature of Astrocyte Diversity. Annu. Rev. Neurosci..

[2] Hertz L., Peng L., Dienel G.A. (2007). Energy metabolism in astrocytes: High rate of oxidative metabolism and spatiotemporal dependence on glycolysis/glycogenolysis. J. Cereb. Blood Flow Metab..

[3] Volterra A., Meldolesi J. (2005). Astrocytes, from brain glue to communication elements: The revolution continues. Nat. Rev. Neurosci..

[4] Belanger M., Allaman I., Magistretti P.J. (2011). Brain energy metabolism: Focus on astrocyte-neuron metabolic cooperation. Cell Metab..

[5] Pestana F., Edwards-Faret G., Belgard T.G., Martirosyan A., Holt M.G. (2020). No Longer Underappreciated: The Emerging Concept of Astrocyte Heterogeneity in Neuroscience. Brain Sci..

[6] Barres B.A. (2008). The mystery and magic of glia: A perspective on their roles in health and disease. Neuron.

[7] Clarke B.E., Taha D.M., Tyzack G.E., Patani R. (2021). Regionally encoded functional heterogeneity of astrocytes in health and disease: A perspective. Glia.

[8] Oberheim N.A., Goldman S.A., Nedergaard M. (2012). Heterogeneity of astrocytic form and function. Methods Mol. Biol..

[9] Buosi A.S., Matias I., Araujo A.P.B., Batista C., Gomes F.C.A. (2018). Heterogeneity in Synaptogenic Profile of Astrocytes from Different Brain Regions. Mol. Neurobiol..

[10] de Freitas L.F., Hamblin M.R. (2016). Proposed Mechanisms of Photobiomodulation or Low-Level Light Therapy. IEEE J. Sel. Top. Quantum Electron..

[11] Batiuk M.Y., Martirosyan A., Wahis J., de Vin F., Marneffe C., Kusserow C., Koeppen J., Viana J.F., Oliveira J.F., Voet T. (2020). Identification of region-specific astrocyte subtypes at single cell resolution. Nat. Commun..

[12] Boisvert M.M., Erikson G.A., Shokhirev M.N., Allen N.J. (2018). The Aging Astrocyte Transcriptome from Multiple Regions of the Mouse Brain. Cell Rep..

[13] Bayraktar O.A., Bartels T., Holmqvist S., Kleshchevnikov V., Martirosyan A., Polioudakis D., Ben Haim L., Young A.M.H., Batiuk M.Y., Prakash K. (2020). Astrocyte layers in the mammalian cerebral cortex revealed by a single-cell in situ transcriptomic map. Nat. Neurosci..

[14] Morel L., Men Y., Chiang M.S.R., Tian Y., Jin S., Yelick J., Higashimori H., Yang Y. (2019). Intracortical astrocyte subpopulations defined by astrocyte reporter Mice in the adult brain. Glia.

[15] Xiong X.Y., Tang Y., Yang Q.W. (2022). Metabolic changes favor the activity and heterogeneity of reactive astrocytes. Trends Endocrinol. Metab..

[16] Mergenthaler P., Lindauer U., Dienel G.A., Meisel A. (2013). Sugar for the brain: The role of glucose in physiological and pathological brain function. Trends Neurosci..

[17] Goyal M.S., Vlassenko A.G., Blazey T.M., Su Y., Couture L.E., Durbin T.J., Bateman R.J., Benzinger T.L., Morris J.C., Raichle M.E. (2017). Loss of Brain Aerobic Glycolysis in Normal Human Aging. Cell Metab..

[18] Vaishnavi S.N., Vlassenko A.G., Rundle M.M., Snyder A.Z., Mintun M.A., Raichle M.E. (2010). Regional aerobic glycolysis in the human brain. Proc. Natl. Acad. Sci. USA.

[19] Wang L., Chaudhari K., Winters A., Sun Y., Liu R., Yang S.H. (2022). Characterizing region-specific glucose metabolic profile of the rodent brain using Seahorse XFe96 analyzer. J. Cereb. Blood Flow Metab..

[20] McCarthy K.D., de Vellis J. (1980). Preparation of separate astroglial and oligodendroglial cell cultures from rat cerebral tissue. J. Cell Biol..

[21] Roy Choudhury G., Winters A., Rich R.M., Ryou M.G., Gryczynski Z., Yuan F., Yang S.H., Liu R. (2015). Methylene blue protects astrocytes against glucose oxygen deprivation by improving cellular respiration. PLoS ONE.

[22] Prah J., Winters A., Chaudhari K., Hersh J., Liu R., Yang S.H. (2019). A novel serum free primary astrocyte culture method that mimic quiescent astrocyte phenotype. J. Neurosci. Methods.

[23] Blodgett A.B., Kothinti R.K., Kamyshko I., Petering D.H., Kumar S., Tabatabai N.M. (2011). A fluorescence method for measurement of glucose transport in kidney cells. Diabetes Technol. Ther..

[24] Tavares G., Martins M., Correia J.S., Sardinha V.M., Guerra-Gomes S., das Neves S.P., Marques F., Sousa N., Oliveira J.F. (2017). Employing an open-source tool to assess astrocyte tridimensional structure. Brain Struct. Funct..

[25] Ruas J.S., Siqueira-Santos E.S., Amigo I., Rodrigues-Silva E., Kowaltowski A.J., Castilho R.F. (2016). Underestimation of the Maximal Capacity of the Mitochondrial Electron Transport System in Oligomycin-Treated Cells. PLoS ONE.

[26] Matyash V., Kettenmann H. (2010). Heterogeneity in astrocyte morphology and physiology. Brain Res. Rev..

[27] Pinto S.S., Gottfried C., Mendez A., Gonçalves D., Karl J., Gonçalves C.A., Wofchuk S., Rodnight R. (2000). Immunocontent and secretion of S100B in astrocyte cultures from different brain regions in relation to morphology. FEBS Lett..

[28] Phillips A.A., Chan F.H., Zheng M.M., Krassioukov A.V., Ainslie P.N. (2016). Neurovascular coupling in humans: Physiology, methodological advances and clinical implications. J. Cereb. Blood Flow Metab..

[29] Magistretti P.J., Allaman I. (2015). A cellular perspective on brain energy metabolism and functional imaging. Neuron.

[30] Prichard J., Rothman D., Novotny E., Petroff O., Kuwabara T., Avison M., Howseman A., Hanstock C., Shulman R. (1991). Lactate rise detected by 1H NMR in human visual cortex during physiologic stimulation. Proc. Natl. Acad. Sci. USA.

[31] Sappey-Marinier D., Calabrese G., Fein G., Hugg J.W., Biggins C., Weiner M.W. (1992). Effect of photic stimulation on human visual cortex lactate and phosphates using 1H and 31P magnetic resonance spectroscopy. J. Cereb. Blood Flow Metab..

[32] Magistretti P.J. (2006). Neuron-glia metabolic coupling and plasticity. J. Exp. Biol..

[33] Magistretti P.J., Pellerin L. (1996). Cellular bases of brain energy metabolism and their relevance to functional brain imaging: Evidence for a prominent role of astrocytes. Cereb. Cortex.

[34] Mason S. (2017). Lactate Shuttles in Neuroenergetics-Homeostasis, Allostasis and Beyond. Front. Neurosci..

[35] Rakic P. (2009). Evolution of the neocortex: A perspective from developmental biology. Nat. Rev. Neurosci..

[36] Azevedo F.A., Carvalho L.R., Grinberg L.T., Farfel J.M., Ferretti R.E., Leite R.E., Jacob Filho W., Lent R., Herculano-Houzel S. (2009). Equal numbers of neuronal and nonneuronal cells make the human brain an isometrically scaled-up primate brain. J. Comp. Neurol..

[37] Molyneaux B.J., Arlotta P., Menezes J.R., Macklis J.D. (2007). Neuronal subtype specification in the cerebral cortex. Nat. Rev. Neurosci..

[38] Consalez G.G., Goldowitz D., Casoni F., Hawkes R. (2020). Origins, Development, and Compartmentation of the Granule Cells of the Cerebellum. Front. Neural Circuits.

[39] Li J., Pan L., Pembroke W.G., Rexach J.E., Godoy M.I., Condro M.C., Alvarado A.G., Harteni M., Chen Y.W., Stiles L. (2021). Conservation and divergence of vulnerability and responses to stressors between human and mouse astrocytes. Nat. Commun..

[40] Rose J., Brian C., Pappa A., Panayiotidis M.I., Franco R. (2020). Mitochondrial Metabolism in Astrocytes Regulates Brain Bioenergetics, Neurotransmission and Redox Balance. Front. Neurosci..

[41] Pacelli C., Adipietro I., Malerba N., Squeo G.M., Piccoli C., Amoresano A., Pinto G., Pucci P., Lee J.E., Ge K. (2020). Loss of Function of the Gene Encoding the Histone Methyltransferase KMT2D Leads to Deregulation of Mitochondrial Respiration. Cells.

[42] Magi S., Piccirillo S., Amoroso S., Lariccia V. (2019). Excitatory Amino Acid Transporters (EAATs): Glutamate Transport and Beyond. Int. J. Mol. Sci..

[43] Mahmoud S., Gharagozloo M., Simard C., Gris D. (2019). Astrocytes Maintain Glutamate Homeostasis in the CNS by Controlling the Balance between Glutamate Uptake and Release. Cells.

[44] McKenna M.C. (2013). Glutamate pays its own way in astrocytes. Front. Endocrinol..

[45] Hoshino M. (2012). Neuronal subtype specification in the cerebellum and dorsal hindbrain. Dev. Growth Differ..

[46] Babot Z., Cristofol R., Sunol C. (2005). Excitotoxic death induced by released glutamate in depolarized primary cultures of mouse cerebellar granule cells is dependent on GABAA receptors and niflumic acid-sensitive chloride channels. Eur. J. Neurosci..

[47] Castilho R.F., Ward M.W., Nicholls D.G. (1999). Oxidative stress, mitochondrial function, and acute glutamate excitotoxicity in cultured cerebellar granule cells. J. Neurochem..

[48] Dolinska M., Zablocka B., Sonnewald U., Albrecht J. (2004). Glutamine uptake and expression of mRNA’s of glutamine transporting proteins in mouse cerebellar and cerebral cortical astrocytes and neurons. Neurochem. Int..

[49] Han B.C., Koh S.B., Lee E.Y., Seong Y.H. (2004). Regional difference of glutamate-induced swelling in cultured rat brain astrocytes. Life Sci..

