# Supplementary material for: Metabolic Heterogeneity of Cerebral Cortical and Cerebellar Astrocytes
Source: Life (Basel). 2023 Jan 8;13(1):184. doi: 10.3390/life13010184 (PMC9860549; doi:10.3390/life13010184)

Supplementary File S1.

The original images of MAP2, in Figure 1 (loading sequence as labeled, control: brain sampleX1; CE mAstro samples X 5; CX mAstro samples X 5).

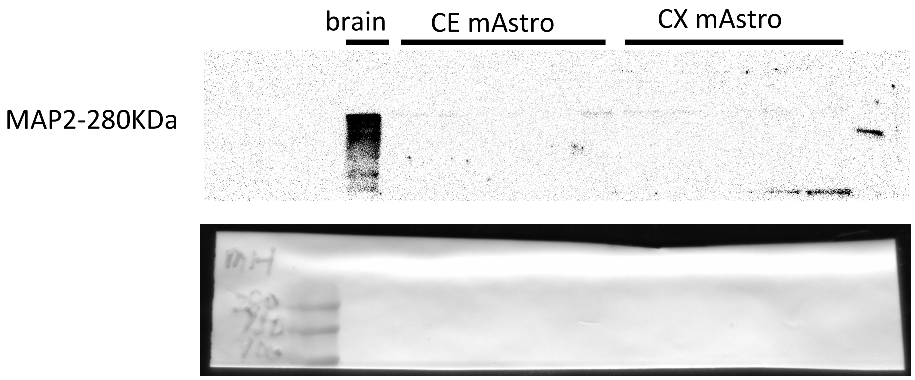

The original images of MBP, in Figure 1 (loading sequence as labeled, control: brain sampleX1; CE mAstro samples X 5; CX mAstro samples X 5).

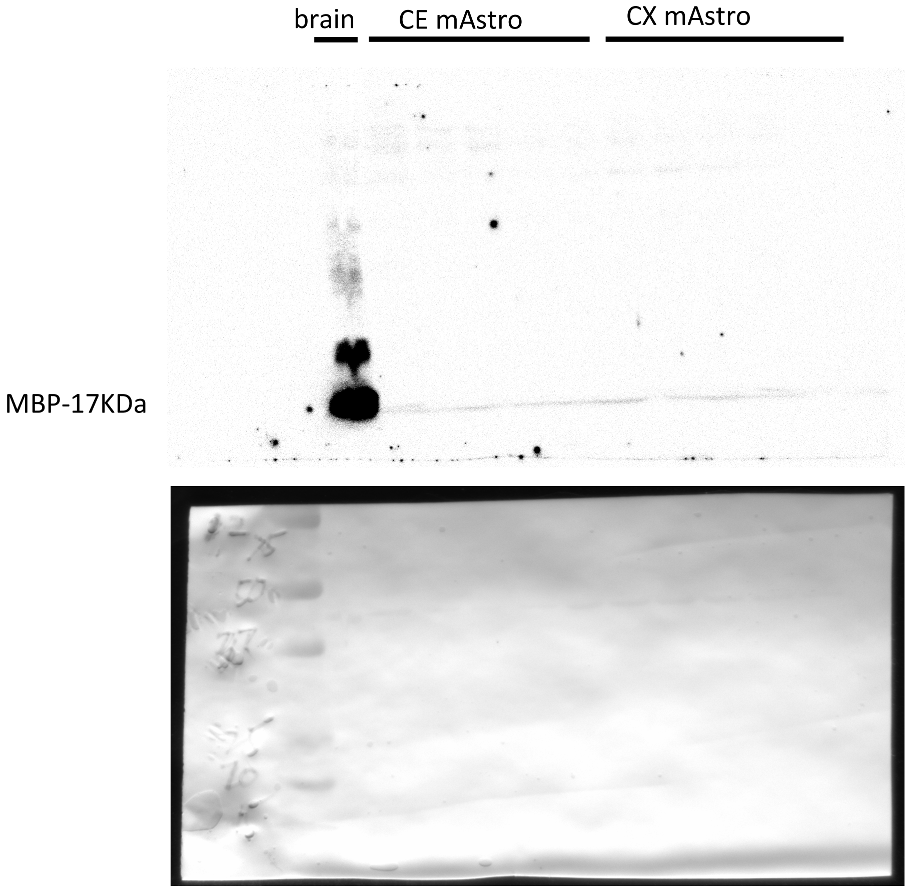

The original images of GAPDH, initial reference of MAP2 and GFAP, in Figure 1 (loading sequence as labeled, control: brain sampleX1; CE mAstro samples X 5; CX mAstro samples X 5).

brain CE mAstro CX mAstro

GAPDH-36KDa

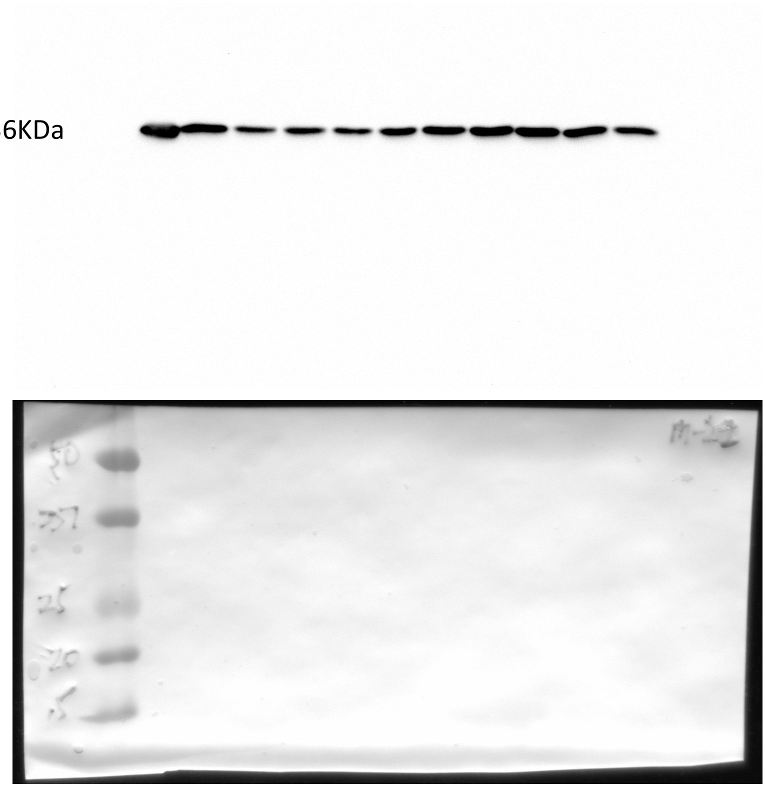

The original images of GFAP, in Figure 1(loading sequence as labeled, control: brain sampleX1; CE mAstro samples X 5; CX mAstro samples X 5).

brain CE mAstro CX mAstro

GFAP-50KDa

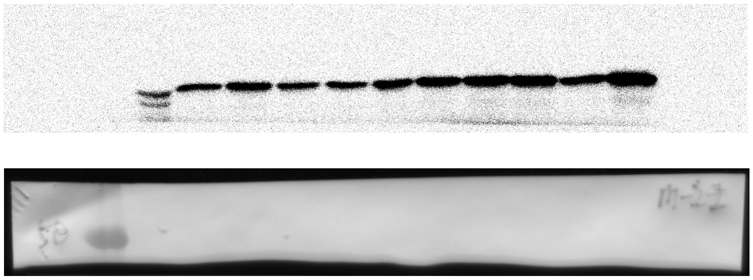

The original images of actin, initial reference of MBP, in Figure 1 (loading sequence as labeled, control: brain sampleX1; CE mAstro samples X 5; CX mAstro samples X 5).

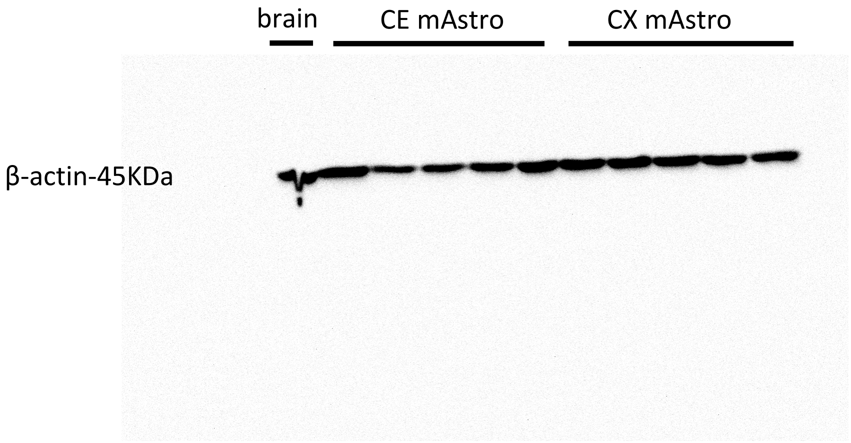

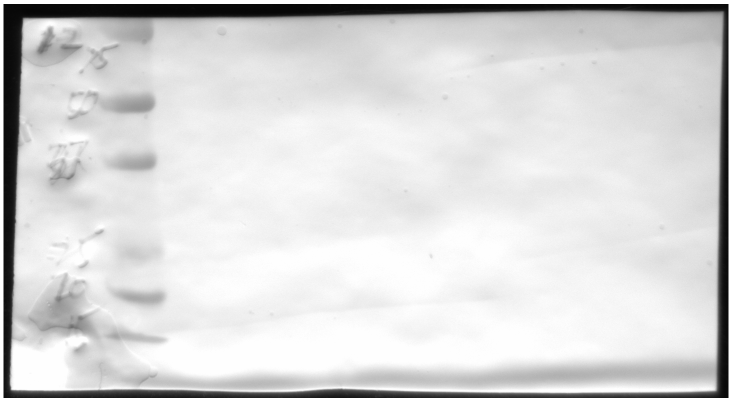

The original images of vimentin, are in Figure 2 (loading sequence as labeled, CX mAstro samples X 3; CE mAstro samples X 4).

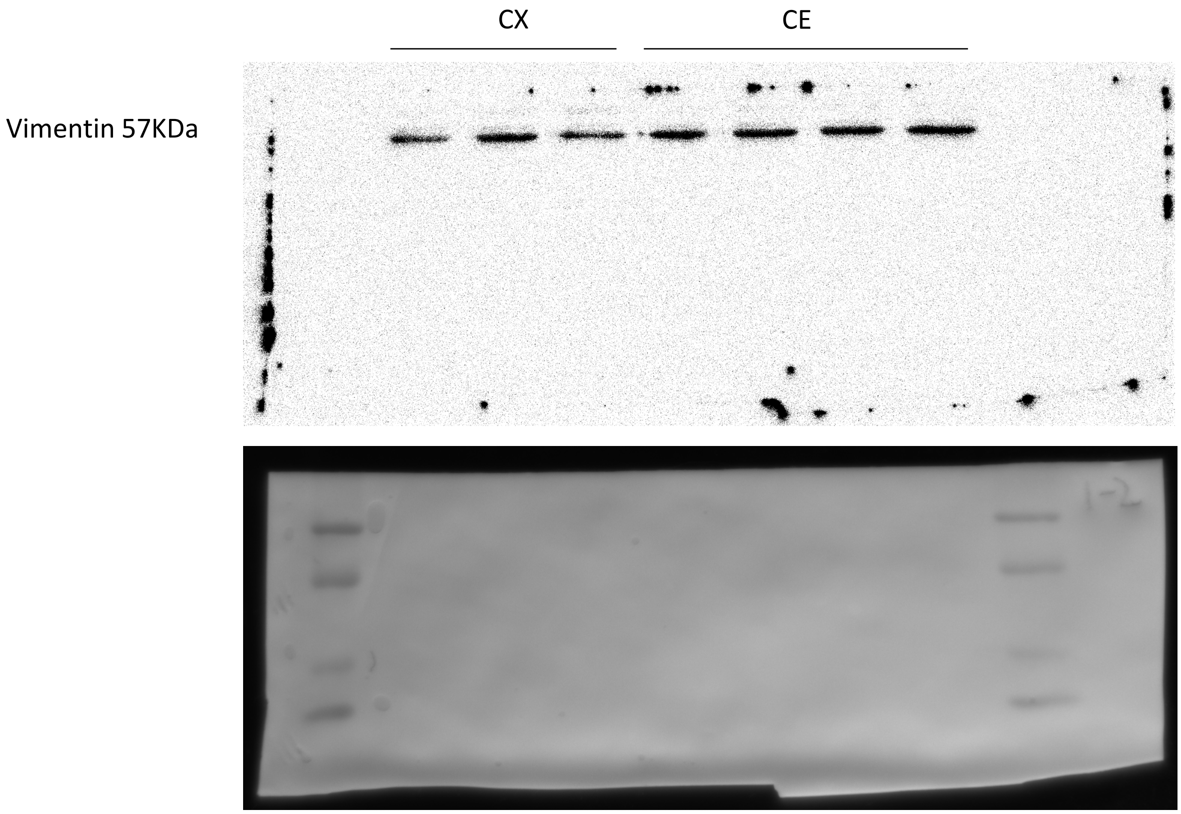

The original images of GAPDH, the initial reference of vimentin, are in Figure 2 (loading sequence as labeled, CX mAstro samples X 3; CE mAstro samples X 4).

CX CE

GAPDH 36 KDa

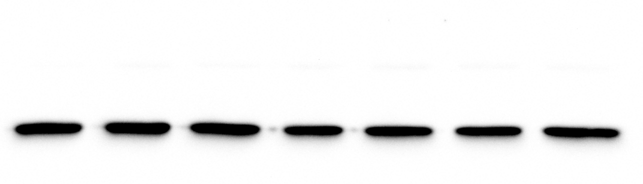

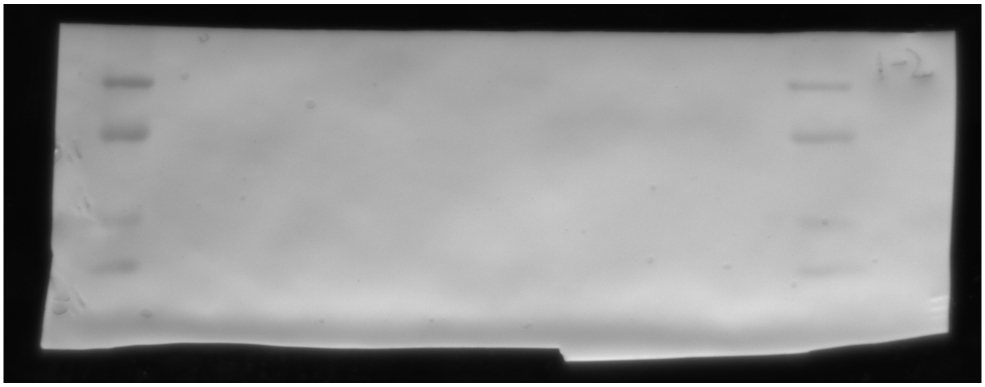

The original images of GFAP, are in Figure 2 (loading sequence as labeled, CX mAstro samples X 3; CE mAstro samples X 4).

CX CE

GFAP 50 KDa

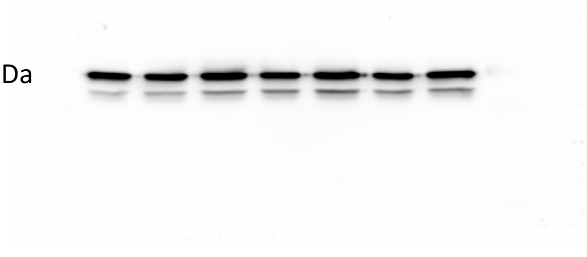

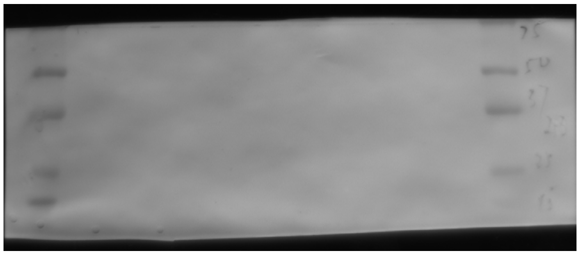

The original images of β-actin, initial reference of GFAP and ALDH1L1 are in Figure 2 (loading sequence as labeled, CX mAstro samples X 3; CE mAstro samples X 4).

This membrane was used to study GFAP expression level first, after the membrane was washing with western blot stripping buffer, the membrane was re-used to investigate the beta-actin. Therefore, the upper band of the image was GFAP and the lower band of the image was beta-actin.

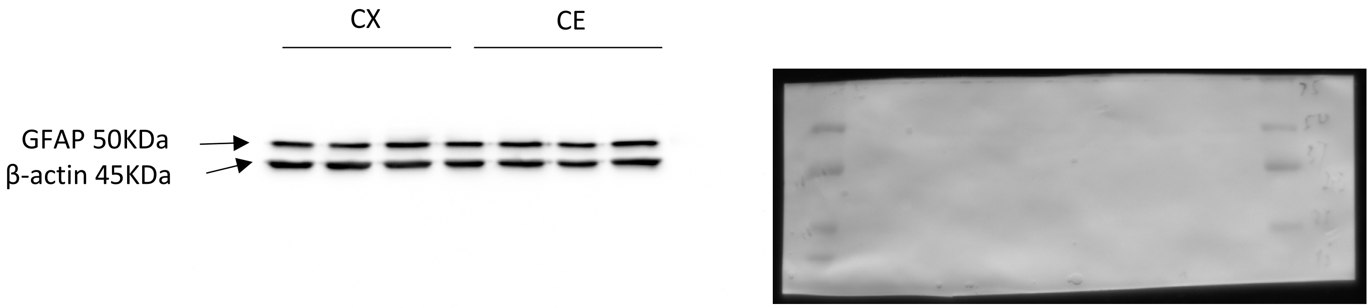

The original images of ALDH1L1, are in Figure 2 (loading sequence as labeled, CX mAstro samples X 3; CE mAstro samples X 4).

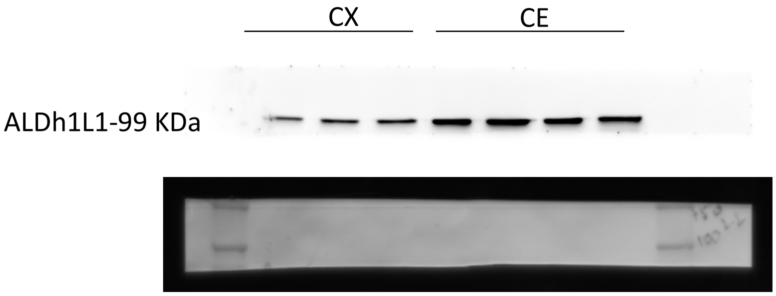

Supplement: Supplementary file 1 [file life-13-00184-s001.zip › life-2075056-supplementary.pdf]
